# ChatGPT’s Role in Improving Education Among Patients Seeking Emergency Medical Treatment

**DOI:** 10.5811/westjem.18650

**Published:** 2024-08-13

**Authors:** Faris F. Halaseh, Justin S. Yang, Clifford N. Danza, Rami Halaseh, Lindsey Spiegelman

**Affiliations:** * University of California, Irvine, School of Medicine, Irvine, California; † Kaiser Permanente San Francisco, Department of Internal Medicine, San Francisco, California; ‡ University of California, Irvine, Department of Emergency Medicine, Irvine, California

## Abstract

Providing appropriate patient education during a medical encounter remains an important area for improvement across healthcare settings. Personalized resources can offer an impactful way to improve patient understanding and satisfaction during or after a healthcare visit. ChatGPT is a novel chatbot—computer program designed to simulate conversation with humans— that has the potential to assist with care-related questions, clarify discharge instructions, help triage medical problem urgency, and could potentially be used to improve patient-clinician communication. However, due to its training methodology, ChatGPT has inherent limitations, including technical restrictions, risk of misinformation, lack of input standardization, and privacy concerns. Medicolegal liability also remains an open question for physicians interacting with this technology. Nonetheless, careful utilization of ChatGPT in clinical medicine has the potential to supplement patient education in important ways.

Population Health Research Capsule What do we already know about this issue?

*Current patient education in the ED is inadequate, often leading to misunderstanding and noncompliance with post-care instructions.*

What was the research question?

*How can the chatbot ChatGPT improve patient education in the ED setting?*

What was the major finding of the study?

*ChatGPT helps triage, clarify discharge, and provide care instructions.*

How does this improve population health?

*ChatGPT can enhance patient education, improving comprehension and adherence to follow-up care, reducing unnecessary ED visits and enhancing overall outcomes.*

## INTRODUCTION

Effective communication and counseling between clinicians and patients is crucial for high-quality healthcare, particularly in the emergency department (ED) where time constraints and evolving diagnostics can complicate discussions about current and post-hospital care. This makes patient education an important gap to address. The urgency and complexity of emergency cases often hinder comprehensive information delivery, leaving patients’ questions unanswered. Inquiries about their condition, treatment options, and prognosis are vital for patient understanding and engagement in decision-making. A systematic review examining the factors that influence the patient experience in the ED highlighted physician-patient communication as the most common factor affecting patient satisfaction.^1^ Addressing these concerns not only boosts satisfaction but also empowers patients, improving health outcomes.

Effective discharge instructions are essential for a patient’s post-emergency care, as ambiguity or inadequate explanation can result in confusion, noncompliance, and subsequent readmission.^2^ ^,^ ^3^ A recent study revealed that 24% of patients do not fully understand their follow-up plan, 64% struggle to comprehend their return-to-ED instructions, and 42% do not receive complete discharge instructions.^3^ Improving discharge communication can enhance transition to follow-up care, thereby improving patient care outcomes and reducing avoidable readmission. In addition to communication challenges, patients may struggle to identify the most appropriate type of care for their conditions. The ED often sees cases that could be more effectively managed in primary care or urgent care settings. About 13–27% of all ED visits are considered non-urgent in nature, resulting in approximately $4.4 billion annual cost.^4^ Identifying factors that contribute to unnecessary emergency care utilization can aid the development of targeted interventions and educational initiatives aimed at redirecting patients to more appropriate avenues of treatment.

Furthermore, increasingly diverse patient populations bring language barriers to the forefront of care in the ED. Patients with limited English proficiency often face obstacles in understanding their diagnoses and treatment plans. One studied showed only 52% of non-English speakers were satisfied with their ED visit, compared to 71% of English speakers.^5^ Strategies to reduce the inequitable impact of language barriers has obvious benefits for patient safety and outcomes in the ED.

One method to address these limitations could be clinical application of a machine learning (ML) large language model (LLM), such as ChatGPT (OpenAI, LP, San Francisco, CA). An ML-based chatbot can learn from a vast array of training data and user interactions to improve performance and efficacy of its delivered communication. In recent performance evaluations, ChatGPT scored above the national average on publicly available renditions of Step 1, Step 2-Clinical Knowledge, and Step 3 of the United States Medical Licensing Exam (USMLE).^6^ The model’s USMLE performance continues to improve with new updates, such as GPT-4, which additionally offers image-analysis capabilities.^7^ Models are also being used to train medical students and prepare them for clerkships.^8^ ^–^ ^10^ These benchmarks demonstrate ChatGPT’s fluency with basic medical information at the knowledge level of a medical school graduate.

Patients often undertake an internet search of medical topics before or after speaking with a physician. However, with a vast array of information and misinformation available on the internet, physicians and other healthcare professionals remain an important interface to source reliable medical knowledge. Here, we evaluate the potential role that LLMs like ChatGPT could have as a personalized educational resource for patients seeking emergency medical treatment.

## CHATGPT: HOW IT WORKS

ChatGPT is a text-based chatbot that facilitates user interaction through natural language. The ML model for the publicly available versions, GPT-3.5 and GPT-4, were trained on written text drawn from a vast spectrum of online databases. Pretraining enables the models to construct a robust linguistic framework. Further conditioning includes assignment-specific adjustments, human feedback that can bolster its conversational abilities, and rigorous testing to ensure resilience against challenging user queries. This process is critical for aligning the model’s output with nuanced human dialogue, enhancing its capacity to provide precise and contextually appropriate responses.^10^

GPT-4 is the latest iteration of this LLM, with an enhanced architecture that not only understands and generates textual content but also interprets and responds to visual input.^11^ Furthermore, while experimental features in GPT-4 include the potential for current internet data, such capabilities have not been broadly released to users. The broader implications of this limitation, including the balance between model autonomy and the necessity for human oversight, are examined further in the section on technical restrictions. Nevertheless, the current capabilities of ChatGPT are an important leap toward a collaborative role that humans and AI systems will share in navigating complex real-world applications.^12^

## PATIENT EDUCATION BENEFITS

The ED is a setting with a particularly high demand for efficient and clear patient education at discharge given the potential reappearance or sequelae of conditions requiring emergent evaluation. However, the ED also often imposes a high degree of constraint on patient-clinician interaction time. Even in the setting of low-acuity conditions, an observational study showed the combined amount of time physicians, nurses, and technicians spent with patients once they are assigned to a bed was about 25% of total patient length of stay.^13^ The factors underlying limited interaction time are multifactorial, but often-cited contributing reasons include patient volume and high load of documentation requirements. Offering a resource that is adaptive and responsive to patient concerns relevant to their specific situation is an important gap in the ED. ChatGPT can provide an interactive modality that is personalized to meet this gap for the healthcare education needs of patients and their clinicians.

### Care-Related Questions

**Figure 1.**
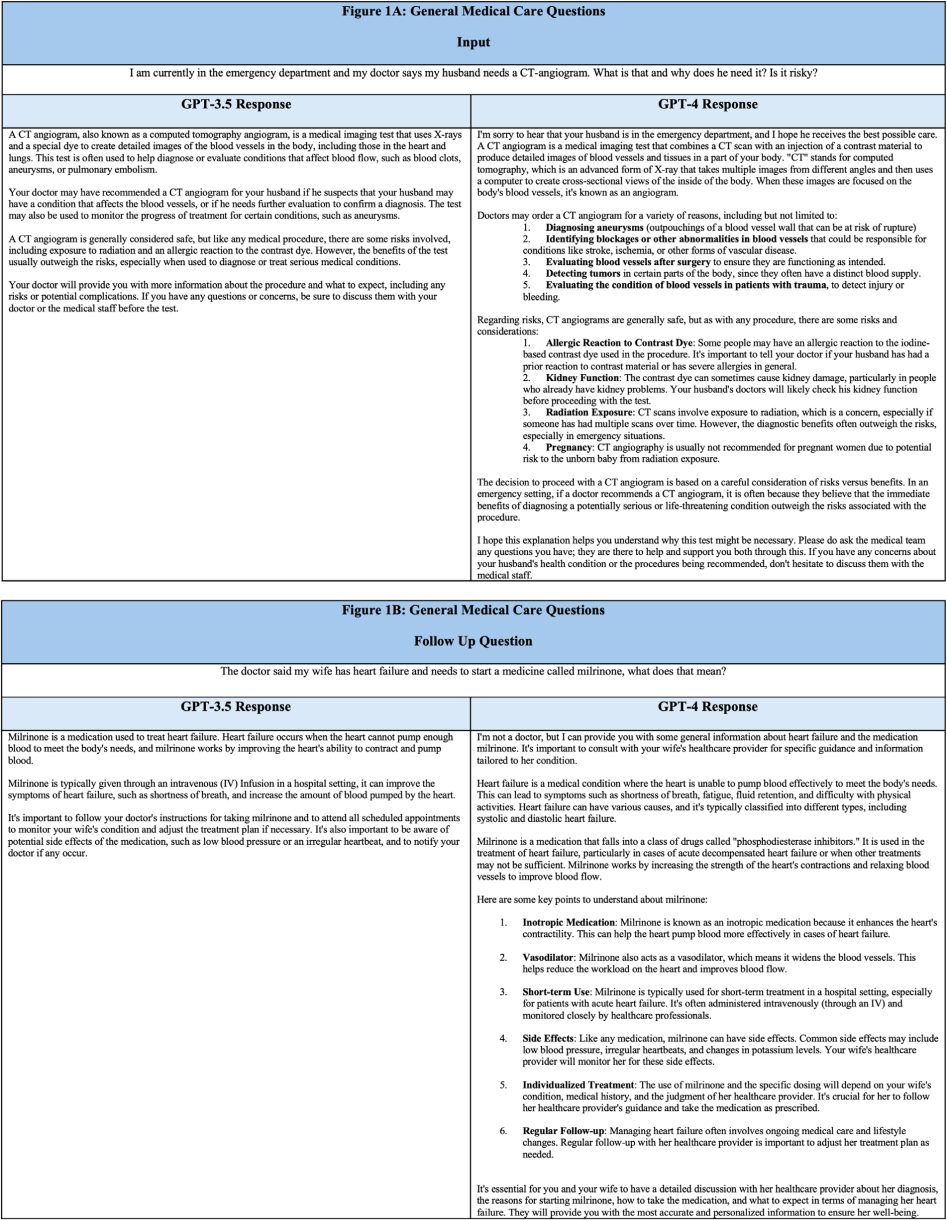
(A–B) Selected Q&A relating to medical care. Inputs are human questions and outputs were generated by ChatGPT.

ChatGPT may offer multiple benefits for patients in the ED. It can quickly give personalized responses to queries on a wide range of healthcare topics. For example, the software can provide purpose- and risk-based information about procedures a patient or their family member may undergo during an ED encounter (Figure 1A). ChatGPT offers background information about the procedure and refers to the care team for further encounter-based questions and concerns. The information provided about a procedure, such as computed tomography angiography in this example, describes an initial depth of response that is likely adequate for a layperson’s introduction. In contrast to conducting a general internet search for the purposes and risks of this procedure, ChatGPT summarizes the pertinent information in a conversational format that is easily accessible to the user.

Another example demonstrates ChatGPT’s sensitivity to slight alterations of meaning when asked about medical conditions and medications. The user first asks a question about the purpose of the medication milrinone for a stated condition of heart failure (Figure 1B). The software’s response explains a short summary for both the condition and the medication.

Given the same set of prompts, there are several differences between GPT-3.5 and GPT-4 outputs. GPT-4 outputs are lengthier, with clearer structure to the answer approach. In these examples, there appears to be a tendency toward itemizing lists. There is also an observed empathetic bent with apparent self-anthropomorphization by starting sentences with “I.” While further study on the end-user impact of this model iteration is warranted, GPT-4 appears to offer more comprehensive information than GPT-3.5 for select healthcare-associated queries (Figure 1).

### Discharge Instructions

**Figure 2.**
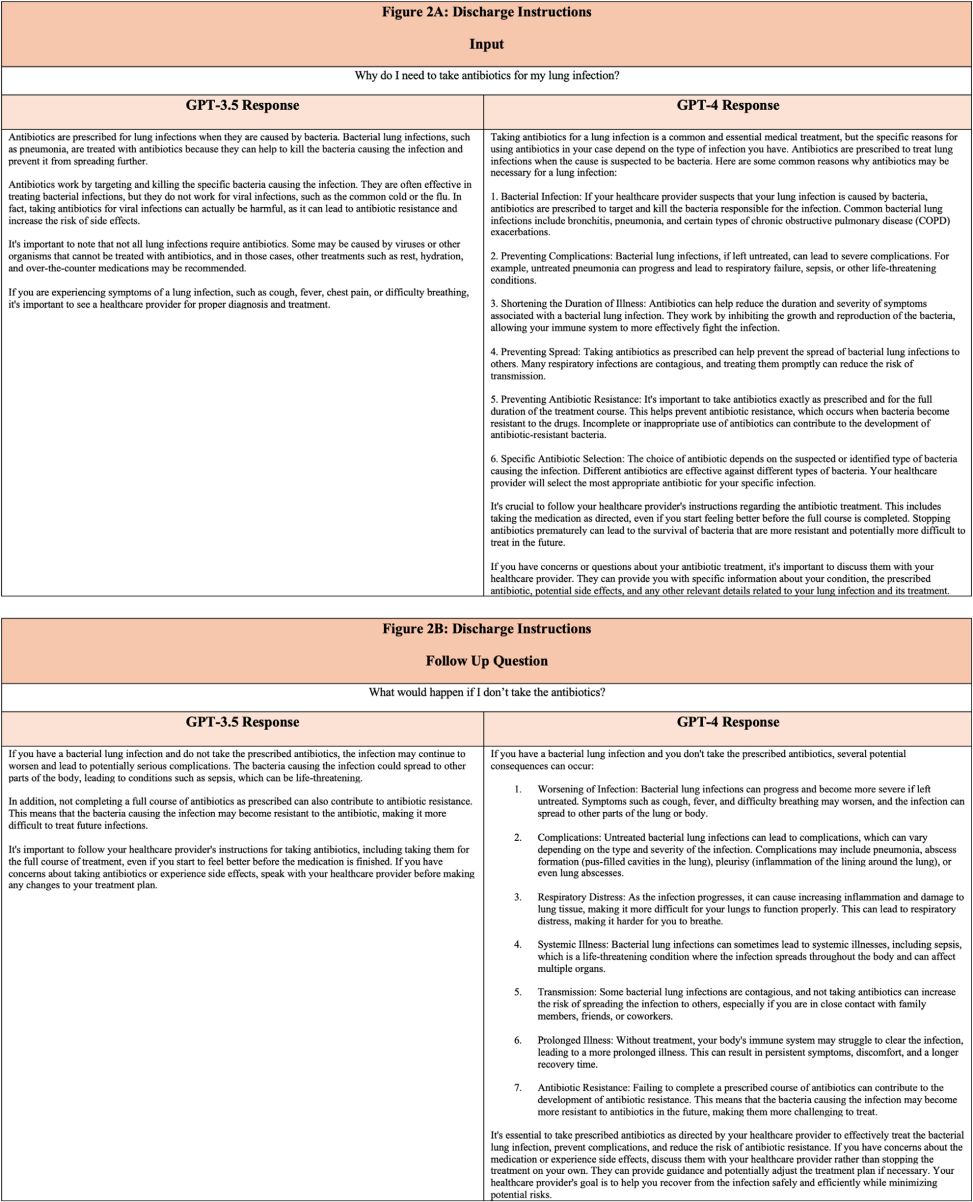
(A–B). Selected questions relating to discharge instructions and ChatGPT output.

Discharge instructions for patients leaving the ED are important for patient safety and satisfaction. However, studies show that patients often do not fully understand the after-care instructions provided to them.^2^ ^,^ ^14^ ^,^ ^15^ Poor adherence to medical advice or follow-up may be attributable in part to a gap in medical understanding. Personalized efforts to increase patient adherence to discharge instructions have focused on staff follow-up via phone call or SMS messaging, but these interventions bear relatively high time and monetary requirements, and do not always provide positive results.^16^ ^–^ ^18^ ChatGPT can be used to improve patient understanding of instructions given by a clinician. Its natural-language interface allows patients to ask questions as if they were conversing with a human (Figure 2A, 2B). Similar tendencies are observed comparing the GPT-4 model to GPT-3.5, with longer answers, increased list creation, and greater use of first- or second-person writing style.

### Seeking Care

Navigating the US healthcare system to determine the appropriate entity from which to seek care is often a significant challenge for patients. The ED remains one of the only settings in which relatively timely evaluation and care is available and is often used as a safety net for patients with no health insurance. However, even patients with insurance and a designated primary care physician sometimes opt for an ED visit due to barriers accessing their physician. Benefits of comprehensive evaluation and services, with imaging, labs, and pharmacy provided in one location is a competitive convenience for patients.^19^ ^,^ ^20^ Due in part to these factors, annual ED utilization rates continue to rise disproportionate to demographic factors, increasing burden on ED resources for conditions that are better addressed in preventive-care settings.^21^

**Figure 3.**
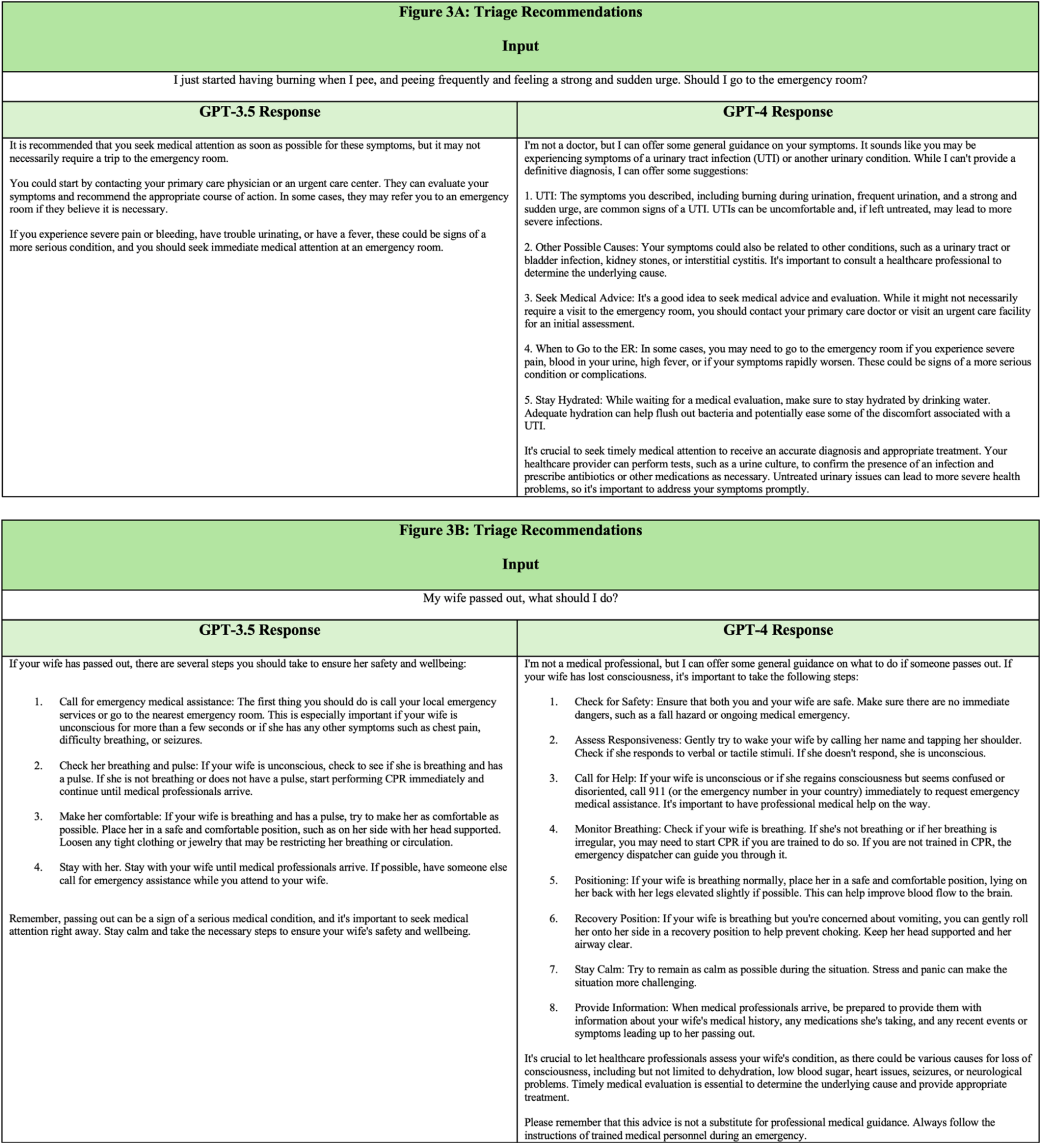
(A–B). ChatGPT input and output. Top output demonstrates ChatGPT triage recommendations and bottom output signifies urgent response instructions.

ChatGPT can provide guidance in seeking urgent vs emergent care. In one example, a patient with symptoms typical of a urinary tract infection asks if a visit to the emergency room is advisable (Figure 3A). The response indicates that care should be sought but suggests urgent care or primary care settings for first-line evaluation, given the stated symptoms. The response also provides examples of more serious symptoms that indicate need for emergent evaluation. Guiding a patient’s decision matrix in a personalized, interactive manner may be an impactful way to encourage them to seek an appropriate level of care. Reducing instances of ED utilization for low-acuity health concerns assists efforts to reduce ED crowding, which can have wide-ranging benefits for healthcare systems by improving patient outcomes.^22^ Future study could examine the usefulness of ChatGPT in triage scenarios, compared to current modalities such as nurse advice phone lines.

ChatGPT can also offer guidance during prehospital emergencies, detailing step-by-step instructions to responders and advising how to seek an appropriate level of care. For example, ChatGPT describes recommended actions upon encountering a syncopal patient (Figure 3B). Prehospital-intervention education geared toward laypersons has been a key focus in public health, particularly for conditions such as cardiac arrest, stroke, and major hemorrhage. While certain practices are best taught in a hands-on demonstration setting, ChatGPT may be a valuable reference resource for laypersons acting in the capacity of an emergency responder.^23^

### Patient Communication

**Figure 4.**
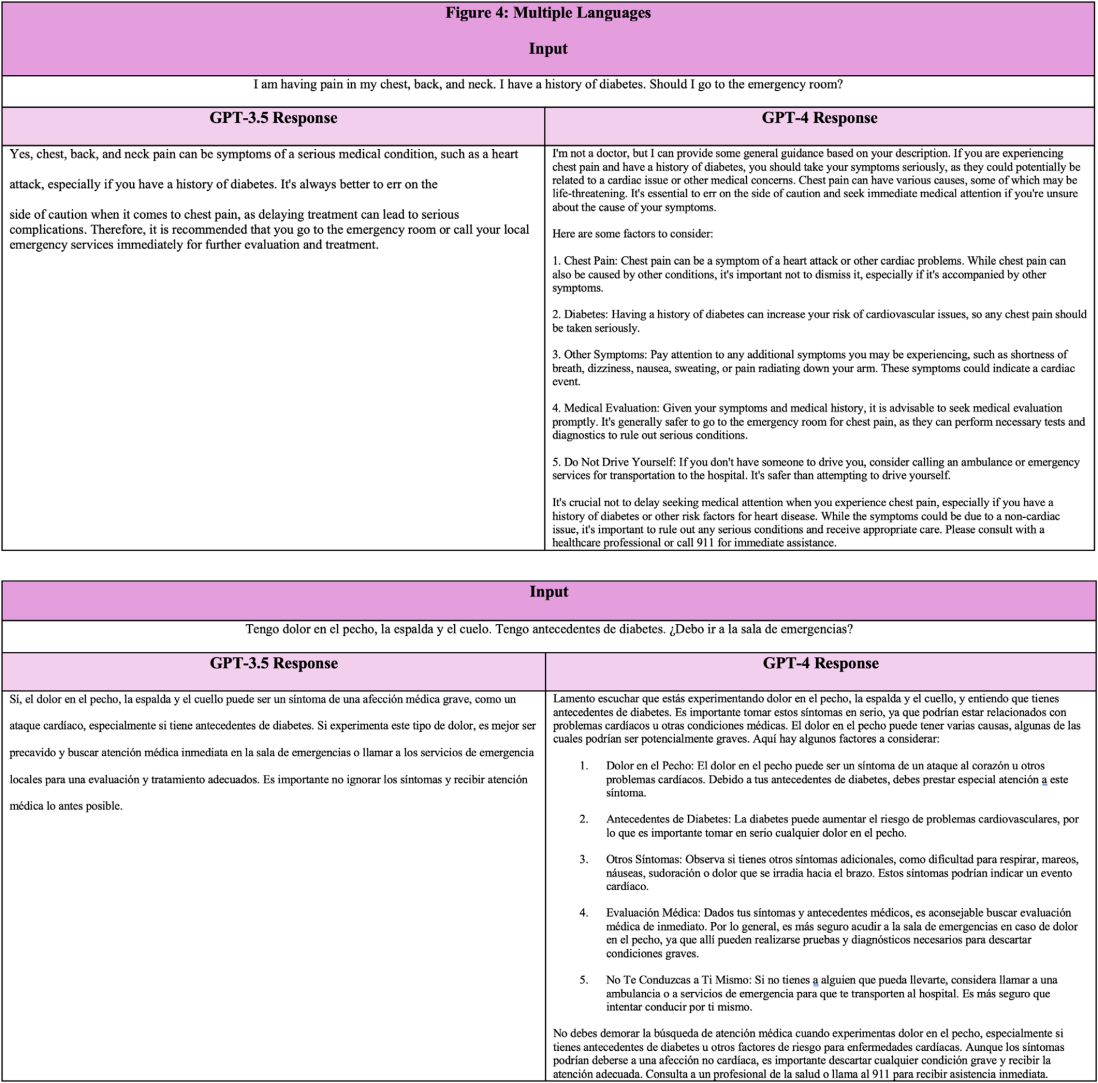
ChatGPT responses in English (top) and Spanish (bottom).

ChatGPT can offer real-time translation for languages including English, Spanish, French, German, Italian, Portuguese, Chinese, Japanese, Korean, Arabic, Dutch, Polish, Russian, and more. While real-time translation is of crucial importance for ED history-taking, translator services are becoming ubiquitous due to the growing popularity of tele-health consultation devices. The benefits of using software with translation capability to address patient questions is apparent in prehospital and post-disposition settings. For example, ChatGPT offers the same response for a question asked first in English, and then in Spanish (Figure 4). Compared to GPT-3.5, GPT-4’s English response takes a more cautious approach to providing medical guidance. However, GPT-4’s Spanish response appears more like GPT-3.5 in the unhedged encouragement for further medical care. This difference may be due to a variety of factors: model language training; translation filters; or underlying differences in training data itself. Further discussion of technical limitations is discussed in the section on technical restrictions. Future studies should investigate the accuracy of ChatGPT translations and compare the results to alternative modalities like certified medical translators, or other text-only modalities like Google Translate, in a clinical healthcare environment.^24^ ^,^ ^25^

### Costs

ChatGPT is currently easily accessible and offers a no-cost user account for the public. A subscription-based upgrade is also offered, which provides quicker response times and early access to new features.^7^ Utilization of ChatGPT to assist patient education for various aspects of care, including understanding health conditions, discharge instructions, and when to seek emergency medical care may save hospital time, money, and resources. Emergency departmemt spending is a growing financial concern on a national level but may be alleviated by reducing the frequency of presentations for preventable causes and avoidable visits.^26^ By providing personal guidance on when to seek emergency care, ChatGPT has the potential to make important contributions to lowering hospital burden.

## RISKS AND LIMITATIONS OF CHATGPT

### Technical Restrictions

Using ChatGPT in emergency medicine carries certain risks. One major concern is that the model can provide different responses to the same or similar questions, which affects its reliability and usefulness. Because ChatGPT is based on a ML algorithm, it may generate different responses to similar questions depending on a variety of factors, such as the order or context of words used as input. In a recent study on ChatGPT’s responses, identical questions regarding cirrhosis and hepatocellular carcinoma were submitted twice and independently graded. ChatGPT was able to produce two similar responses 90.48% of the time. Although this number may be regarded as high reproducibility, it raises concerns over the potential consistency of offering medical advice to laypersons. Although most instances will provide the user with the same information, ChatGPT’s responses are based on probability.^27^ Future studies should analyze this variability to determine the impact on response appropriateness for the user.

In addition to output variability, there is also a potential for differences in user comprehension. This underscores another risk: the potential for differences in comprehension and interpretation of the information provided to laypersons. Although this limitation is common among many different technologies, these differences in comprehension can lead to potentially adverse outcomes for patients. Given these considerations, it is crucial to exercise caution and apply critical thinking when using ChatGPT as a source of information in healthcare interactions. It is essential to be aware of the potential for developing overconfidence in ChatGPT’s recommendations, and the risk for anchoring on a specific diagnosis or impression for both patients and clinicians.

Response variability and interpretation of responses can impact the consistency and reliability of this model. It is important for clinicians and patients to be aware of these risks, and to use ChatGPT as a supplementary tool rather than a primary source of information. Therefore, ongoing improvements in the technology and continued training of the algorithm should be pursued. Efforts to incorporate current medical literature into the training data could enhance the reliability and relevance of ChatGPT’s responses. However, it remains essential to recognize the dynamic nature of clinical practice and the importance of relying on evidence-based guidelines to ensure patient safety and well-being.

### Misinformation

Ensuring accurate and truthful information is crucial when using ChatGPT, as misinformation can have serious consequences for patient care. ChatGPT’s training data is limited to information available before 2021. Medical knowledge and practices have evolved since this knowledge cutoff point, and ChatGPT may reference information that is outdated or no longer applicable to current medical practices. According to the ChatGPT study for cirrhosis and hepatocellular carcinoma, ChatGPT’s answers contained a mix of correct and incorrect information. The percentage of responses that were classified as having both correct and incorrect or outdated answers was 22% in basic knowledge, 33% in diagnosis, 25% in treatment, 18% in lifestyle, and 50% in preventive medicine.^28^ However, while this study highlights potential limitations of ChatGPT’s accuracy in responding to questions about hepatocellular carcinoma, it is important to recognize that further research is needed to comprehensively evaluate the accuracy of ChatGPT’s responses across all medical domains. Physicians and patients must remain cautious when using ChatGPT and seek confirmation from other reliable sources before making decisions based on the information provided.

Another concern is the model’s opaque algorithm, which can generate plausible sounding but inaccurate or nonsensical answers, a phenomenon known as “hallucinations”.^29^ Evidence of this can be seen in fabricated sources when ChatGPT is asked to provide references for a given response.^30^ ChatGPT’s training may have included scientific literature citation datasets, but its generative algorithm does not allow for one-to-one data source matching, resulting in a response that provides completely fabricated sources. Patients may be less likely to question the accuracy of ChatGPT’s responses if they are presented with a source and simply assume that the information has been verified. This can ultimately lead to patients making decisions that are not in their best interest and may even result in harm.

There is currently unavoidable risk of misinformation when using ChatGPT for patient education in emergency medicine. To mitigate this risk, it is important to manage expectations and risk thresholds. Physicians and patients should be advised of the risks and benefits of this technology. Additionally, regular evaluation and improvement of the model will help minimize the risk of inaccuracies and misunderstandings.

### Privacy and Security

Ensuring patient privacy and security is another concern when using ChatGPT for patient encounters. Unauthorized access to protected health information (PHI) can lead to identity theft, insurance fraud, and other types of harm to the patient. For physicians, unauthorized disclosures violate the Health Insurance Portability and Accountability Act (HIPAA) and can lead to disciplinary action, loss of licensure, and legal liability. OpenAI, the creators of ChatGPT, have stated that data used with ChatGPT will remain secured by default, with an opt-out option to share data for research and quality improvement. Nevertheless, major corporations such as Verizon and JPMorgan & Co. have restricted employees from accessing ChatGPT due to concerns over possible data breaches.^31^ Sensitive healthcare information could be at a similar risk.

While the public availability of the ChatGPT application programming interface makes it easy to integrate into websites and applications, it also raises concerns about the security and privacy of patient information. As the use of artificial intelligence (AI) chatbots become more widespread, it is increasingly important to ensure that these technologies are used in a way that protects patient privacy and complies with regulations such as HIPAA. Integrating ChatGPT into a HIPAA-compliant framework may help address these concerns. Healthcare technology leaders must take necessary measures to protect PHI.

### Medicolegal and Other Ethical Consideration

Integration of ChatGPT into emergency medicine presents a complex landscape of medicolegal and ethical implications.^32^ We have previously discussed limitations with the software: potential for misdiagnosis and delayed treatment is a significant concern. Artificial intelligence systems, while advanced, may not always accurately interpret patient symptoms, which is dependent upon the quality of user input and the software’s understanding. This has potential to influence patient outcomes, and there are numerous situations that could do the same.

Consider the following hypothetical, ethical scenarios:

• ChatGPT recommends against seeking care, which results in a harmful or life-threatening patient outcome.
• ChatGPT provides false information to a patient.
• Patients inadequately advised on the risks/benefits of ChatGPT misinterpret its analysis.
• Patient PHI is accessed during a data breach or during the performance improvement process.
• ChatGPT provides information that is not up to date or conflicts with current guidelines.

In the unfortunate circumstance where one or more of these events occur and legal action is taken, who should be held responsible—OpenAI, clinicians, or both? This is a current challenge that necessitates further interdisciplinary discussion between stakeholders.^33^ Nonetheless, there are steps that must be taken to help minimize risk for all parties involved. For example, there should be restrictions placed on LLMs prior to official implementation in the field. Whether through legislation or an independent body, ChatGPT must adhere to regulatory standards that ensure HIPAA-compliance and informed consent.^34^ Physicians and OpenAI must also work toward education on potential risks of the software. Physicians and other healthcare professionals should also implement legal forms and liability waivers into the care process to ensure protection in instances where these regulations fail.

## TRANSITIONING INTO CLINICAL STUDIES

The next major step that must be taken is to validate ChatGPT’s efficacy and safety in clinical settings.^32^ One of the major barriers that researchers may face is the ever-changing updates to the software, which is also a limitation of this paper. Not only is ChatGPT continually being updated, but the protocols by which fine-tuning, updates, and further training occur are confidential.^10^ This inherently makes ChatGPT difficult to study. However, potential future studies with ChatGPT are many and should assess the accuracy, safety, readability, and semantic analysis of the software. One such future study could consider the efficacy of patient triage for ChatGPT and triage nurse phone calls. Future studies should also investigate the cost benefit of implementing such a system into ED workflow, either for triage, discharge instructions, or both. This should be corroborated by examining potential algorithm bias in the real world.^32^ ^,^ ^34^ ^,^ ^35^

## THE PHYSICIAN’S ROLE IN CHATGPT

As stated previously, ChatGPT cannot replace a physician. Although OpenAI has made significant strides in developing a software that communicates in a more human-like, empathetic manner compared to previous chatbots, current technology still lacks the oversight and nuance offered by a human. This technology cannot replace an in-depth history, physical exam, or clinical reasoning. However, given the current rate of progress on these technologies, it would be naive to consider a future independent of technology-assisted patient encounters. For this reason, physicians and healthcare professionals must be involved in research and development oversight to ensure accurate data is available on these platforms, and that these technologies are being developed with the right intentions. Physicians should also continue to be informed on AI developments, so that they can play a proactive role in educating patients on the benefits, limitations, and liability of the software. Further studies must also examine the legal implications for physicians, patients, and OpenAI, as ChatGPT and healthcare continue to intersect.

## CONCLUSION

This paper describes the use of ChatGPT as an educational resource for patients seeking emergency medical treatment. Although limitations such as technical issues, misinformation risk, lack of input standardization, and privacy concerns exist, this software offers compelling benefits for patient education. The software can answer questions specific to patients and their presentations, allowing for a personalized educational resource. ChatGPT can also clarify discharge instructions, help triage urgent vs emergent conditions, and it can respond in multiple languages. Physicians must understand these benefits and limitations to best guide patients and conduct further research in new AI technologies.
